# Cancer mortality in Common Mental Disorders: A 10-year retrospective cohort study

**DOI:** 10.1007/s00127-022-02376-x

**Published:** 2022-11-17

**Authors:** Federico Chierzi, Elisa Stivanello, Muriel Assunta Musti, Vincenza Perlangeli, Paolo Marzaroli, Francesco De Rossi, Paolo Pandolfi, Alessio Saponaro, Luigi Grassi, Martino Belvederi Murri, Angelo Fioritti, Domenico Berardi, Marco Menchetti

**Affiliations:** 1 Department of Mental Health and Substance Abuse, Local Health Trust of Bologna, Bologna, Italy; 2 Department of Public Health, Local Health Trust of Bologna, Bologna, Italy; 3 Department of Biomedical and Neuromotor Sciences, University of Bologna, Bologna, Italy; 4 Institute of Psychiatry, Department of Neuroscience and Rehabilitation, University of Ferrara, Ferrara, Italy; 5 General Directorate of Health and Social Policies, Emilia-Romagna Region, Bologna, Italy

**Keywords:** Public mental health, Cancer, Mortality, Comorbidity, Physical illness

## Abstract

**Purpose:**

Individuals with Common Mental Disorders (CMDs) may have a higher cancer mortality. The purpose of this study was to examine cancer-related mortality among patients with CMDs and verify which cancer types are predominantly involved.

**Methods:**

We used the Regional Mental Health Registry of the Emilia-Romagna region, in Northern Italy to identify patients aged ≥ 18 years who received an ICD 9-CM diagnosis of CMDs (i.e., depressive and neurotic disorders) over a 10 year period (2008–2017). Information on cause of death was retrieved from the Regional Cause of Death Registry. Comparisons were made with data from the regional population without CMDs.

**Results:**

Among 101,487 patients suffering from CMDs (55.7% depression; 44.3% neurotic disorders), 3,087 (37.8%) died from neoplasms. The total standardized mortality ratio (SMR) was 1.82 (95% CI 1.78–1.86) while the SMR for all neoplasms was 2.08 (95% CI 2.01–2.16). Individuals of both genders, with both depressive and neurotic disorders had a higher risk of death from almost all cancers compared with the regional population.

**Conclusion:**

Patients with CMDs have considerably higher cancer mortality risk than the general population. Higher mortality was observed for a broad range of cancers associated with different aetiologies. It is imperative to promote cancer awareness, prevention and treatment for people with CMDs.

**Supplementary Information:**

The online version contains supplementary material available at 10.1007/s00127-022-02376-x.

## Introduction

Depression and anxiety disorders–Common Mental Disorders (CMDs)—are highly prevalent [1] and are the important causes of disability [2]. The association between CMDs and chronic medical illnesses such as cardiovascular diseases, diabetes, and cancer may even increase the risk of premature death [3].

The relationship between depressive–anxiety disorders and neoplasms has been increasingly studied in the last two decades [4]. Not surprisingly, studies of patients with cancer found a high prevalence of depression and anxiety disorders [5]. Moreover, CMDs have been suggested to be predictors of newly diagnosed neoplasms [6] and are associated with a worsening of cancer outcomes, including mortality [7]. Therefore, it has been hypothesized that depression and anxiety may play an etiologic role in the development and progression of neoplasms [8].

Epidemiological studies examining the relationship between CMD and cancer have yielded mixed and inconclusive findings. Some studies did not find any associations between depression and increased cancer mortality [9], while others suggested that only a small and non-significant relationship exists [10]. A recent meta-analysis of 51 studies found that people affected by depression and anxiety have a significantly higher risk (RR 1.21, 95% CI 1.16–1.26) of neoplasm mortality than psychologically healthy individuals [4]. However, included studies were characterized by high clinical and methodological heterogeneity [4]. In particular, most of them recruited patients with depressive and anxiety symptoms (e.g., so-called psychological distress syndrome) [8–11]; only a minority included patients with a verified clinical diagnosis [12]. Moreover, the bulk of the existing literature concerns the survival of oncological patients with newly diagnosed CMDs, usually enrolled from hospital cancer units [13–16]. Very few studies included patients from specialist mental healthcare settings [12]. Using the latter recruitment strategy would improve the reliability of the diagnosis, as in that setting it would be made by a psychiatrist according to standardized criteria. Some additional limitations of the published literature include a predominant focus on depressive disorders [11, 17–19], even if the few studies including people with anxiety disorders found they had an increased risk of cancer death. Specifically, mortality rates were similar to those estimated for depressive disorders [13, 20]. Given that anxiety disorders are also widespread, their association with cancer mortality deserves a greater attention [21]. Finally, most studies examined cancer-related mortality without differentiating between site specific-cancers [11, 22, 23]. The association between CMDs and cancer may vary according to cancer type [4] because of different biological and behavioral mechanisms and risk factors.

The aim of this study was to explore cancer-related mortality in a large sample of individuals with CMDs, including both depression and anxiety, and to consider the mortality for specific types of cancer.

## Methods

This registry-based, retrospective cohort study examined a large dataset of patients diagnosed and treated by mental health specialists over a 10-year period (2008–2017) in the Mental Health Departments of Emilia-Romagna (ER), a region in northern Italy with about 4.5 million inhabitants.

In ER, patients suffering from psychiatric disorders that are reactive to cancer can be referred to different clinical services. A psycho-oncology service has been operating for many years in the region [24]. Specific psychological support services are usually integrated within hospital cancer units [25] and home palliative care units [26]. In addition, a consultation liaison psychiatric service for primary care is available to support general practitioners, usually treating individuals with milder clinical conditions. More severe cases requiring multidisciplinary care, social support, and/or intensive psychiatric rehabilitation are usually referred to secondary mental healthcare services [27]. ER has a community-based mental healthcare system based on eight Mental Health Departments (MHD), one for each Local Health Trust, as detailed in previous studies [28, 29]. This study included all patients aged 18 years or over who accessed the regional MHD and received a diagnosis of a CMD, namely depression or neurotic disorders (the ICD-9 equivalent of DSM anxiety, trauma, and somatic related disorders) during the period 1 January 2008 to 31 December 2017. Psychiatric diagnoses were defined according to the International Classification of Disease, Ninth Revision, Clinical Modification (ICD-9 CM). For the diagnostic codes included in our study see Table 1 in Online Resources.

**Table 1:** Demographic characteristics of the study population at their first access to the Mental Health Department

	Common mental disorders		Depression		Neurotic disorders	
	*n*	%	*n*	%	*n*	%
Total						
Gender	101,487	100.00	56,489	100.00	44,998	100.00
Women	64,620	63.67	37,054	65.60	27,566	61.26
Men	36,867	36.33	19,435	34.40	17,432	38.74
Age group. years						
18–24	7,405	7.30	2,359	4.18	5,046	11.21
25–44	35,745	35.22	17,197	30.44	18,548	41.22
45–64	35,786	35.26	22,188	39.28	13,598	30.22
65–74	12,165	11.99	7,877	13.94	4,288	9.53
75–84	8,733	8.61	5,838	10.33	2,895	6.43
85 +	1,653	1.63	1,030	1.82	623	1.38
Citizenship						
Italian	91,411	90.07	51,225	90.68	40,186	89.31
Non italian	10,076	9.93	5,264	9.32	4,812	10.69
Residency						
Urban^a^	37,612	37.06	20,641	36.54	16,971	37.72
Rural	63,875	62.94	35,848	63.46	28,027	62.28

^a^This includes all residents in municipalities with more than 60,000 inhabitants

The regional Mental Health Registry was used to identify study participants and retrieve demographic data (age, gender, citizenship, and residency), psychiatric diagnosis, and the date of first referral. The registry was developed for administrative, clinical, and epidemiological purposes; it contains information on psychiatric diagnoses, dates of visits, and treatments (pharmaceutical, psychological, and rehabilitative) of all inpatients and outpatients who accessed the MHD. Demographic features, as well as diagnoses, are routinely recorded at the first clinical evaluation and then constantly updated by clinicians; all data are collected together via an information system at the regional level. Residents outside the ER region and patients who had accessed the MHD before 2008 were excluded.

To retrieve information on deaths (dates and causes of death), the Mental Health Registry was linked with the Regional Cause of Death Registry (for the years 2008–2017). For this purpose, we assigned a single numeric anonymous identity code to subjects registered in all regional health archives. The Regional Cause of Death Registry covers all deaths of the regional population and contains information about socio-demographic characteristics, date, location, and circumstances of death. The main cause of death is routinely collected immediately after death. Within 1 year, all deaths are coded according to the ICD-10 classification (International Classification of Disease, tenth revision, Clinical Modification). The cause of death that arose first and/or had the greatest influence on death is chosen, following the criteria of the World Health Organization [30] Both registries undergo regular systematic quality control.

For this study, we considered deaths for all causes and for total neoplasms, further divided into site-specific malignant neoplasms identified by specific ICD-10 codes (Table 2 in Online Resources). Patients who changed their residence to another region according to the Regional Population Archive were considered lost to follow-up because of the impossibility of ascertaining their life status. The regional population was used as the comparator group. The Regional Population Archive provided aggregated data on the age and gender composition of this population. The study was approved by the local Ethical Committee (N. 341/2019).

**Table 2:** Number of deaths and proportional mortality of all causes of death, all neoplasms, and site-specific malignant neoplasms. Comparison between regional and study population in the whole sample

	Regional population		Common mental disorders		Depression		Neurotic disorders	
	*n*	%	*n*	%	*n*	%	*N*	%
All causes of deaths	484,102	100.00	8,172	100.00	5,484	100.00	2,688	100.00
All neoplasms	143,311	29.60	3,087	37.78	2,068	37.71	1,019	37.91
Site-specific malignant neoplasms								
Oesophagus	1,330	0.27	40	0.49	31	0.57	9	0.33
Stomach	9,149	1.89	167	2.04	112	2.04	55	2.05
Colon, rectosigmoid junction, rectum, anus	15,043	3.11	288	3.52	210	3.83	78	2.90
Liver, gallbladder, biliary tract	9,144	1.89	161	1.97	107	1.95	54	2.01
Pancreas	9,834	2.03	205	2.51	139	2.53	66	2.46
Larynx	996	0.21	39	0.48	23	0.42	16	0.60
Trachea, bronchus, and lung	28,492	5.89	622	7.61	427	7.79	195	7.25
Melanoma	1,413	0.29	24	0.29	17	0.31	7	0.26
Breast	9,610	1.99	286	3.50	183	3.34	103	3.83
Uterus (cervix, corpus, unspecified)	2,338	0.48	65	0.80	41	0.75	24	0.89
Ovary and other female genital organs	3,087	0.64	105	1.28	70	1.28	35	1.30
Prostate	5,694	1.18	87	1.06	58	1.06	29	1.08
Kidney, renal pelvis, ureter and unspecified urinary Organs	4,420	0.91	86	1.05	61	1.11	25	0.93
Bladder	4,962	1.02	103	1.26	64	1.17	39	1.45
Meninges, brain and other parts of CNS^a^	3,460	0.71	97	1.19	53	0.97	44	1.64
Lymphoid, hematopoietic and related tissue	12,032	2.49	272	3.33	188	3.43	84	3.13

^a^Central Nervous System

### Statistical analyses

We calculated frequency of death for all causes, for neoplasms, and for each site-specific malignant neoplasm in the whole study population and separately by diagnosis (depression or neurotic disorders). We calculated the standardized mortality ratio (SMR) with 95% confidence intervals (CI) to compare the risk of mortality between the regional and the study population. The SMR is the ratio between the number of observed deaths and the number of expected deaths as if the study population had the same gender and age rates (the latter grouped in classes) as the regional population. An SMR equal to 1 indicates the number of observed deaths is equal to the number of expected deaths, an SMR greater than 1 indicates that there are more deaths than expected and an SMR lower than 1 indicates that the deaths are lower than expected. The SMR was also calculated by gender (controlling for age class only), type of cancer, and psychiatric diagnosis in cases in which there were more than three observed deaths. The differences between observed and expected deaths were considered statistically significant if the 95% CI of the SMR did not include the value of one. Person-years were used as the denominator and were calculated as the difference between the date of the first access to a Mental Health Department and either the 31 December 2017, the date of death or the date of moving to another region, whichever was earlier.

## Results

The cohort consisted of 101,487 subjects suffering from common mental disorders, 56,489 from depressive disorders, and 44,998 from neurotic disorders. Table 1 shows the main demographic characteristics of the population at their first admission to the Mental Health Department.

During the study period, there were 8,172 deaths for all causes and 3,087 deaths for in the study population, with a higher proportion of cancer mortality compared to the regional population (37.8 vs. 29.6%, p = 0.001).

As shown in Table 2, the proportion of deaths due to neoplasms does not differ between neurotic disorders and depression. As site-specific neoplasms are concerned, the five most frequent causes of death are: cancer of the trachea/bronchus and lung, lymphoid and hematopoietic neoplasms, cancer of the colon/rectum/anus, pancreatic and breast cancer in both the regional population and in the population with CMDs. They appear in the same ranking except for breast cancer that is more frequent in the CMDs population than in the regional population.

The standardized all-cause mortality ratio was 1.8 times (95% CI 1.78–1.86) higher in patients with CMDs than in the regional population and the mortality for neoplasms was more than double in the CMDs population (SMR 2.08, 95% CI 2.01–2.16). The mortality ratio was higher in patients with depression than in patients with neurotic disorders (SMR 2.18, 95% CI 2.09–2.28 vs. 1.91, 95% CI 1.79–2.03) (Table 3).

**Table 3:** SMR for all causes of death, for all neoplasms, and for site-specific malignant neoplasms. Whole study sample

	Common Mental Disorders			Depression			Neurotic disorders		
	SMR^a^	95% CI^b^		SMR	95% CI		SMR	95% CI	
All causes of deaths	1.82	1.78	1.86	1.89	1.84	1.94	1.69	1.63	1.76
All neoplasms	2.08	2.01	2.16	2.18	2.09	2.28	1.91	1.79	2.03
Site-specific malignant neoplasms									
oesophagus	3.00	2.14	4.08	3.70	2.52	5.26	1.81	0.83	3.44
Stomach	1.85	1.58	2.15	1.93	1.59	2.32	1.70	1.28	2.21
Colon, rectosigmoid junction, rectum, anus	1.89	1.68	2.13	2.15	1.87	2.46	1.44	1.13	1.79
Liver, gallbladder, biliary tract	1.73	1.48	2.02	1.80	1.47	2.17	1.62	1.22	2.12
Pancreas	1.95	1.69	2.23	2.05	1.72	2.42	1.76	1.36	2.24
Larynx	4.31	3.06	5.89	4.06	2.57	6.09	4.72	2.70	7.66
Trachea, bronchus and lung	2.17	2.00	2.34	2.34	2.13	2.57	1.86	1.61	2.14
Melanoma	1.59	1.02	2.37	1.82	1.06	2.91	1.22	0.49	2.52
Breast	2.23	1.98	2.51	2.22	1.91	2.57	2.26	1.84	2.74
Uterus (cervix, corpus, unspecified)	2.07	1.60	2.64	2.02	1.45	2.74	2.17	1.39	3.22
Ovary and other female genital organs	2.44	1.99	2.95	2.51	1.96	3.17	2.30	1.60	3.19
Prostate	1.97	1.58	2.43	2.07	1.57	2.68	1.78	1.19	2.56
Kidney, pelvis, ureter, and unspecified urinary organs	2.02	1.61	2.49	2.24	1.72	2.88	1.62	1.05	2.39
Bladder	2.34	1.91	2.84	2.27	1.75	2.90	2.47	1.76	3.37
Meninges, brain, and other parts of CNS^c^	2.43	1.97	2.96	2.14	1.60	2.79	2.91	2.12	3.91
Lymphoid, hematopoietic, and related tissue	2.20	1.94	2.47	2.37	2.04	2.73	1.89	1.51	2.34

^a^SMR: Standardized Mortality Ratio

^b^CI: Confidence Interval

^c^Central Nervous System

Males showed a higher mortality ratio than females when considering all-cause mortality (SMR 2.04, 95% CI 1.98–2.11 vs SMR 1.67, 95% CI 1.62–1.72), but not when considering only cancer-related deaths (Table 4). Table 4 shows that the SMR was greater than 1 in all cases except for colon/rectal/anal cancer in women with neurotic disorders. The SMR was always significant except for melanoma in males with any common mental disorders, cancer of the oesophagus in neurotic patients (of both genders), and cancer of the bladder in women with depression.

**Table 4:** SMR for all causes of death, for all neoplasms, and for site-specific malignant neoplasm. Separately by gender

Females									
	Common Mental Disorders			Depression			Neurotic disorders		
	SMR^a^	95% CI^b^		SMR	95% CI		SMR	95% CI	
All causes of deaths	1.67	1.62	1.72	1.74	1.68	1.80	1.53	1.45	1.61
Total neoplasms	2.02	1.92	2.11	2.13	2.01	2.26	1.80	1.65	1.96
Site-specific malignant neoplasms									
Oesophagus	2.44	1.26	4.25	3.12	1.50	5.74	–	–	–
Stomach	1.85	1.48	2.28	1.93	1.48	2.49	1.68	1.11	2.45
Colon, rectosigmoid junction, rectum, anus	1.70	1.44	2.00	2.07	1.71	2.48	0.99	0.66	1.42
Liver, gallbladder, biliary tract	1.51	1.17	1.90	1.62	1.20	2.14	1.28	0.78	1.97
Pancreas	1.94	1.61	2.31	2.00	1.59	2.47	1.82	1.30	2.48
Larynx	5.46	2.50	10.00	5.54	2.03	12.00	–	–	–
Trachea, bronchus, and lung	2.25	1.99	2.54	2.50	2.16	2.87	1.79	1.41	2.25
Melanoma	2.07	1.18	3.36	2.25	1.12	4.02	1.76	0.57	4.11
Breast	2.24	1.99	2.52	2.22	1.91	2.57	2.27	1.85	2.75
Uterus (cervix, corpus, unspecified)	2.07	1.60	2.64	2.02	1.45	2.74	2.17	1.39	3.22
Ovary and other female genital organs	2.44	1.99	2.95	2.51	1.96	3.17	2.30	1.60	3.19
Prostate	–	–	–	–	–	–	–	–	–
Kidney, pelvis, ureter, and unspecified urinary organs	2.37	1.73	3.19	2.70	1.86	3.79	1.75	0.87	3.13
Bladder	1.66	1.07	2.48	1.57	0.88	2.59	1.85	0.85	3.52
Meninges, brain, and other parts of CNS^c^	2.04	1.50	2.71	1.83	1.21	2.67	2.39	1.46	3.69
Lymphoid, hematopoietic, and related tissue	1.96	1.65	2.32	2.10	1.71	2.56	1.69	1.21	2.29

Males									
	Common Mental Disorders			Depression			Neurotic disorders		
	SMR	95% CI		SMR	95% CI		SMR	95% CI	
All causes of deaths	2.04	1.98	2.11	2.12	2.04	2.21	1.91	1.81	2.01
Total neoplasms	2.17	2.06	2.29	2.25	2.11	2.40	2.04	1.86	2.22
Site-specific malignant neoplasms									
Oesophagus	3.33	2.21	4.81	4.06	2.52	6.21	2.16	0.87	4.45
Stomach	1.84	1.46	2.29	1.93	1.44	2.52	1.71	1.13	2.47
Colon, rectosigmoid junction, rectum, anus	2.14	1.80	2.52	2.25	1.82	2.76	1.96	1.45	2.58
Liver, gallbladder, biliary tract	1.96	1.58	2.41	1.98	1.50	2.57	1.93	1.34	2.70
Pancreas	1.97	1.56	2.45	2.14	1.61	2.80	1.69	1.10	2.47
Larynx	4.05	2.73	5.78	3.71	2.16	5.94	4.60	2.45	7.87
Trachea, bronchus, and lung	2.11	1.89	2.34	2.23	1.95	2.53	1.91	1.58	2.28
Melanoma	1.09	0.47	2.14	1.35	0.49	2.93	–	–	–
Breast	–	–	–	–	–	–	–	–	–
Uterus (cervix, corpus, unspecified)	–	–	–	–	–	–	–	–	–
Ovary and other female genital organs	–	–	–	–	–	–	–	–	–
Prostate	1.97	1.58	2.43	2.07	1.57	2.68	1.78	1.19	2.56
Kidney, pelvis, ureter, and unspecified Urinary organs	1.74	1.26	2.35	1.87	1.24	2.71	1.53	0.84	2.56
Bladder	2.68	2.12	3.34	2.64	1.95	3.49	2.74	1.85	3.91
Meninges, brain, and other parts of CNS^c^	2.97	2,20	3,91	2.58	1.68	3.77	3.56	2.28	5.29
Lymphoid, hematopoietic, and related tissue	2.51	2.10	2.98	2.74	2.21	3.37	2.14	1.55	2.88

^a^SMR: standardized mortality ratio

^b^CI: confidence interval

^c^Central nervous system

SMR was not computed in categories with less than 4 observed cases

**Fig. 1:**
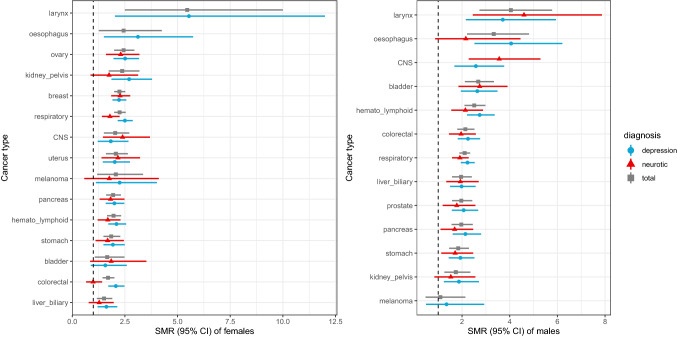
Standardized mortality ratio (SMR) and 95% confidence interval (CI) for site-specific malignant neoplasm by gender

As shown in Tables 3 and 4, the SMR for larynx cancer was significantly higher than the SMR for all neoplasms in depressed patients and in neurotic patients, in particular for males. A higher SMR was also registered for cancer of the oesophagus in depressed patients. Moreover, standardized mortality was also notably high for central nervous system (CNS) cancers in neurotic patients as well as for ovarian cancer, especially among patients with depression. A lower SMR was registered for melanoma in both depressed and neurotic patients and for the cancer of the liver and biliary tract. With the exception of cancer of the larynx, no significant differences in the SMR were observed between most cancer subtypes (see Fig. 1).

## Discussion

Our study investigated cancer-related mortality among people with CMDs, recruiting its sample from the public mental healthcare setting. Our main finding is that the risk of dying from cancer was substantially higher in people with CMDs than in the regional population. Higher mortality was found for almost all neoplasms in patients affected by depressive and neurotic disorders, although there was some variability in the additional risk depending on the type of tumor. In particular, larynx and oesophagus cancer were the main causes of cancer mortality in both genders; while the SMR for ovarian cancer was particularly elevated among women, and the SMR for CNS cancers was highest among men.

Previous studies found greater mortality from cancer in people with depressive disorders [11, 18, 19, 22, 31], but they reported risk estimates (SMRs from 1.3 to 1.8) that are significantly lower than our estimates. According to a French study, which recruited participants through a survey [11], the risk of dying from cancer was elevated only for women with depression but not for men. We found a significantly increased mortality risk in both genders. Other large prospective-cohort studies investigated the association between depressive and anxiety symptomatology, jointly defined as psychological distress, and cancer-related mortality, finding a modest but statistically significant increase [8, 32]. However, this finding was not replicated in other prospective studies [9, 10, 24]. Such heterogeneity may be explained by differences in study designs, sample sizes, and other methodological aspects, including the way in which the samples were selected. The present study was conducted on a large cohort of mental healthcare service users with relatively severe and recurrent illness, while surveys with random sampling methods recruited a smaller and probably less severely ill sample [11, 31]. Moreover, previous studies mostly ascertained the presence of CMDs using psychometric tests [9, 33], often self-administered [8, 10, 32], rather than using clinical diagnoses. The detection of lower risk, or failure to detect increased mortality may be due to the inclusion of individuals with sub-threshold syndromes, as confirmed by a recent meta-analysis [4].

Interestingly, we found that not only patients with depression but also those with neurotic disorders were at higher risk of dying from neoplasms, suggesting a possible link between cancer and the whole spectrum of CMDs. Depressive and anxiety disorders may share genetic diathesis [34]. A common genetic background has also been demonstrated between depression and some types of cancer [35, 36]. A possible mediator of this common genetic background could be underlying personality traits (such as neuroticism and extraversion) [34]. These have been shown to predispose to anxiety and depression [34], as well as to increased exposure to risk factors for cancer [37] and to cancer mortality [38]. These hypotheses need to be further explored, considering possible confounders and mediators of this relationship.

Another finding of our study is that excess mortality spanned almost all types of neoplasms. People affected by depression were at elevated risk of death from all cancer types; among patients with neurotic disorders, only the SMR for oesophageal cancer and melanoma did not reach statistical significance. These findings do not necessarily reflect a causal effect between CMDs and cancer-related mortality, but they are in line with two previous studies showing that depression and anxiety could be associated with shorter survival in patients with a broad range of cancers [14, 20]. A further study [17] found that depressed individuals had an increased cancer-related mortality irrespective of cancer site, but the leading causes of death were due to breast and lungs cancers. We detected higher mortality in people with CMDs for a wide spectrum of cancers: both characterized by high heritability and those closely related to lifestyle; both neoplasms targeted by screening programs and those not targeted, which often have a late diagnosis; and also in cancers with a variety of prognoses. Thus, multiple putative pathophysiological pathways might link CMDs with cancer-related mortality at the biological, psychological, and social levels.

First, neoplasm incidence and progression could be enhanced in people with CMDs. Several studies indicate that depression, anxiety, and all the aforementioned cancers may share a common biological liability induced by chronic inflammation and immune system function impairment [39]. For instance, dysregulation of the hypothalamic–pituitary–adrenal (HPA) axis as well as altered autonomic responses have been suggested as mediators of the vulnerability to hormone-related neoplasms (e.g., breast and ovarian cancers) due to chronic inflammation [40, 41]. In addition, CMDs may lead to unhealthy lifestyles [42]. The high propensity of patients with CMDs to smoke [43] could increase the risk of developing several types of cancers, especially those of the respiratory tract such as laryngeal cancer [44], and urothelial carcinomas such as bladder cancer [45]. Poor quality diet [46] and high alcohol consumption [47] may facilitate the process of oesophageal [48] and laryngeal [44] carcinogenesis in people with CMDs. Finally, tricyclic antidepressants and SSRIs should also be mentioned, as few studies found a link between the use of these drugs and an increased risk of breast [49] and ovarian cancer [50].

Second, cancer diagnosis and treatment could be influenced by comorbidity with CMDs. Growing evidence suggests that people with depression and anxiety disorders receive poorer levels of care compared to the general population [51]. This has been associated with a more advanced staging at cancer diagnosis and higher mortality, particularly from some neoplasms such as breast and colorectal cancer [52]. Moreover, studies conducted on insurance databases found inadequate treatment even after cancer diagnosis. In particular, men with prostate cancer and comorbid depression were less likely to undergo radical surgery and radiotherapy [53]compared with non-depressed men. Women suffering from depression prior to cancer diagnosis had a lower likelihood of receiving definitive treatment for breast cancer, such as surgery or chemotherapy [54]. In both cases, a diagnosis of depression was associated with poorer survival. Furthermore, depressive symptoms such as a loss of interest and initiative may delay or hinder treatment plans [55]. Several studies showed that having depression predicted non-adherence to adjuvant hormonal therapy in breast cancer survivors [55, 56].

The major strengths of our study are the large-scale cohort design, the considerable follow-up, the independently and consistently collected data on both depressive and anxiety disorders, and causes of death. In addition, we were able to investigate mortality for all specific types of cancer.

However, the following limitations should be taken into account. First, it could be argued that some patients developed CMDs in reaction to the diagnosis of cancer, thus inflating the magnitude of the association, but in fact being a case of reverse causality [15]. In addition, some patients may have been referred to the mental health service because of cancer-related psychiatric symptoms (misdiagnosis). This could be true, for example, for CNS cancers [57], whose clinical onset may be initially masked by depression or anxiety syndromes. We did not have information on the temporal relationship between cancer and CMD onset. Collaborative care may have favored referrals from general practitioners to mental health services after cancer diagnosis [27]. However, there is unlikely to be a high proportion of patients referred by primary care services in our study. Specific psycho-oncologic and psychological services within the general hospital usually provide care for the majority of patients with primary cancer and secondary mental disorders, without referring them to a mental health setting [24]. Other individuals with CMDs receive care from general practitioners, private psychiatrists or—unfortunately—remain undiagnosed and untreated.

Second, we did not account for the competing risks of other causes of death (such as cardiovascular diseases or suicide) which may have precluded the occurrence of death from neoplasms among one of the two groups. Our cancer mortality estimates could thus be affected by this bias.

Third, we had no information on potential confounders such as other physical comorbidities, socio-economic conditions or environmental exposure (e.g., lifestyles, substance use etc.) that might also affect mortality among individuals with CMDs. Further studies are required to investigate this potential source of bias.

Finally, anxiety and depression disorders may co-occur; thus, the epidemiological correlation between anxiety and cancer mortality could be partly inflated by comorbid depression [58]. As we considered only the main psychiatric diagnosis, this cannot be completely ruled out.

## Conclusions

Patients with CMDs have an excess mortality from cancer compared to the general population. Our study shows that both depressive and anxiety disorders are associated with an increased risk of death from a broad range of site-specific neoplasms. Further research is needed to investigate the relationship between the onset and the course of CMDs and cancer mortality, taking into account competing causes of death. There are many potential pathophysiological mechanisms underlying this association, which should also be analyzed. In the meantime, we highlight a need for closer cooperation between primary care physicians, mental health services, and oncology specialists. Patients with CMDs need increased clinical attention, focused cancer prevention initiatives, and careful monitoring to achieve early diagnosis and appropriate treatment.

## Supplementary Information

Below is the link to the electronic supplementary material.

Supplementary file1 (PDF 203 KB)

## Data Availability

Data of the study will be shared on reasonable request.
