# Supplementary material for: Cancer mortality in Common Mental Disorders: A 10-year retrospective cohort study
Source: Soc Psychiatry Psychiatr Epidemiol. 2022 Nov 17;58(2):309–18. doi: 10.1007/s00127-022-02376-x (PMC9922233; doi:10.1007/s00127-022-02376-x)
Supplement: Supplementary file 1 — Supplementary file1 (PDF 203 KB) [file 127_2022_2376_MOESM1_ESM.pdf]

Title: Cancer mortality in Common Mental Disorders: A 10-year retrospective cohort study

Journal: Social Psychiatry and Psychiatric Epidemiology

Authors: Federico Chierzi; Elisa Stivanello; Muriel Assunta Musti; Vincenza Perlangeli; Paolo Marzaroli; Francesco De Rossi; Paolo Pandolfi; Alessio Saponaro; Luigi Grassi; Martino Belvederi Murri; Marco Menchetti; Angelo Fioritti; Domenico Berardi

Corresponding Author: Professor Marco Menchetti. marco.menchetti3@unibo.it. Department of Biomedical and Neuromotor Sciences, University of Bologna, Bologna Italy; Department of Mental Health and Substance Abuse, Local Health Trust of Bologna, Bologna Italy

Table 1. ICD 9 CM for the classification of the diagnostic group

Diagnostic Group	***ICD9-CM*[a]**
**Depression**	296, 296.2*, 296.3*, 296.82, 296.9*, 298.0, 300.4, 309.0, 309.1, 309.1, 311
**Neurotic disorders**	300* (escl. 300.4), 306*, 307.4*, 307.8*, 307.9, 308*, 309.2* (escl. 309.28), 309.8*, 316

a. ICD: International Classification of Diseases

Table 2. ICD-10 codes for causes of death; all neoplasm and site-specific malignant neoplasm.

**Causes of deaths**	**ICD-10[a] codes**
**All neoplasms**	C. D1-D4
**Site specific malignant neoplasms**	
oesophagus	C15
stomach	C16
colon, rectosigmoid junction, rectum, anus	C18-C21
liver, gallbladder, biliary tract	C22-C24
pancreas	C25
larynx	C32
trachea, bronchus and lung	C33-C34
melanoma	C43
breast	C50
uterus (cervix, corpus, unspecified)	C53-C55
ovary and other female genital organs	C56-C57
prostate	C61
kidney, renal pelvis, ureter and unspecified urinary organs	C64-C66, C68
bladder	C67
meninges, brain and other parts of CNS	C70-C72
lymphoid, hematopoietic and related tissue	C81-C96

a. ICD: International Classification of Diseases
